# Disseminating brief video-based mental health interventions on Instagram: Reach, engagement, and real-world implementation

**DOI:** 10.1016/j.invent.2026.100990

**Published:** 2026-08-12

**Authors:** Shilat Haim-Nachum, Andrés Martin, Doron Amsalem

**Affiliations:** a School of Social Work, Tel Aviv University, Tel Aviv, Israel; b The Sagol School of Neuroscience, Tel Aviv University, Tel Aviv, Israel; c Department of Psychiatry, Columbia University Irving Medical Center, New York, NY, USA; d New York State Psychiatric Institute, New York, NY, USA; e Yale Child Study Center, Yale School of Medicine, New Haven, CT, USA

**Keywords:** Childhood trauma, Video-intervention, Stigma, Help-seeking, Social media

## Abstract

**Objective:**

Childhood trauma (CT) profoundly impacts mental health. Negative attitudes and stigma surrounding mental health and trauma often deter survivors from seeking help. Previous research demonstrated that a brief video intervention significantly reduced stigma and enhanced openness to treatment among CT survivors. Given social media's pervasive role in young people's lives, Instagram offers a promising avenue for delivering evidence-based interventions. This study aimed to test the feasibility and acceptability of disseminating this intervention via Instagram.

**Methods:**

Two consecutive campaigns targeted U.S. youth aged 18–24. The first campaign featured a human-narrated personal story video shown to reduce stigma among CT survivors, alongside a psychoeducational control video without social-contact elements. The second campaign aimed to replicate these findings and explore the feasibility of additional videos, including artificial intelligence (AI)-generated videos. We assessed key engagement metrics: impressions (total views), reach (unique viewers), link clicks (engagement with treatment resources), and cost-per-click (CPC) for recruitment.

**Results:**

The campaigns generated approximately 997,000 impressions, reaching 387,068 Instagram users, resulting in 6417 link clicks. Using click-through rate (CTR), compared via two-proportion z-tests, as the primary comparative metric, the human-narrated intervention significantly outperformed the control videos on a per-impression basis in both campaigns, and on a per-reach basis against the ‘About Me’ control in Campaign 2, but not on a per-reach basis in Campaign 1 or against the psychoeducational control in Campaign 2. AI-generated videos showed significantly higher per-reach CTR than the human-narrated intervention in Campaign 2.

**Conclusion:**

These findings suggest Instagram's potential to disseminate cost-effective interventions previously shown to reduce negative attitudes toward CT and encourage youth to seek help. Future studies should examine long-term effects on treatment-seeking behaviors in different populations.

Clinical trial number: not applicable.

## Highlights

• Instagram can effectively deliver stigma-reducing interventions for trauma survivors.
• Two campaigns reached over 380,000 young adults with a brief stigma intervention.
• The intervention increased engagement with mental health resources.
• AI-generated videos matched or exceeded engagement with human-narrated videos.

## 1 Introduction

Childhood trauma (CT), which can involve physical, emotional, or sexual abuse or neglect, is experienced by approximately one in four individuals in the US (Brown et al., 2023) with other studies suggesting substantially higher rates, likely reflecting underreporting and differences in how CT is defined and assessed (Merrick et al., 2018). CT poses substantial risk to development across multiple domains of functioning, including cognitive, emotional, and behavioral, with psychopathology one of the most salient sequelae (Teicher and Samson, 2016; Lippard and Nemeroff, 2020). Trauma-exposed children are three times more likely than others to develop psychopathology, including depression, anxiety, and posttraumatic stress disorder (PTSD) (Hughes et al., 2017; KA et al., 2012; Li et al., 2016). In fact, nearly half of psychological disorders can be attributed to CT experiences (Zeanah and Humphreys, 2018).

Despite its prevalence and debility, survivors of CT often avoid seeking care (Kantor et al., 2022). This avoidance can stem from several factors, such as limited resources, access to mental health services, or fears of treatment (Kantor et al., 2017). Moreover, young survivors may not recognize their symptoms as a mental health problem or know where or how to access effective mental health services (Radez et al., 2020; Gulliver et al., 2010). One primary barrier to treatment-seeking is stigma, societal prejudices or negative stereotypes about one's own identity or experiences (Clement et al., 2015; Hofmann et al., 2023; Schomerus et al., 2021). Stigma instills shame, blame, and fear of rejection, exacerbating reluctance to seek help (Hofmann et al., 2023; Schomerus et al., 2021). It correlates with diminished quality of life and heightened symptom severity across various mental disorders (Schomerus et al., 2021; Corrigan and Rao, 2012). Youth survivors of CT, particularly those who have experienced interpersonal trauma such as abuse and neglect, are often prone to negative perceptions, including blame, guilt and shame, which contribute to the development of stigma (Schomerus et al., 2021; Kim and Cicchetti, 2010). Research has shown that stigma mediates the relationship between CT experiences and various forms of psychopathology, highlighting the importance of developing and disseminating interventions aimed at reducing stigma and increasing treatment-seeking intentions (Haim-Nachum et al., 2025).

Social media platforms are increasingly used by youth to seek mental health services and support (Kauer et al., 2014). These platforms can play a significant role in facilitating access to resources and community support (Office of the Surgeon General, 2023). They can promote help-seeking behaviors among youth, potentially serving as access points to mental health care (Pretorius et al., 2019). Several digital strategies have surfaced to involve young individuals, a noteworthy development given that approximately half of mental health disorders emerge during youth (Solmi et al., 2021). Additionally, social media platforms provide avenues for crucial information, social backing, and outlets for self-expression, which is especially crucial for marginalized groups facing barriers to mental health resources (Schueller et al., 2019).

Yet, alongside its benefits, social media use also carries risks, such as exposure to content that could reinforce stigma, or negative perceptions, surrounding mental health treatments, potentially hindering youth's efforts to seek help. Social media users often lack access to evidence-based content and are not equipped to identify misinformation or harmful content (Zarocostas, 2020), particularly in an era where much of the content is generated by artificial intelligence (AI; Guo et al., 2024). Comprehensive research is needed to develop content that promotes engagement with mental health services while safeguarding young users from potential negative impacts.

Research suggests that the use of sympathetic narratives — stories that humanize individuals with mental illness — may paradoxically increase stigma, emphasizing the need for robust research (Kennedy-Hendricks et al., 2016). We have previously used crowdsourcing platforms as a testing lab to examine the efficacy of social contact-based brief video interventions in reducing stigma and increasing help-seeking intentions across various populations (Amsalem et al., 2022). These interventions featured protagonists sharing their experiences with depression (Amsalem and Martin, 2022; Amsalem et al., 2024) and childhood trauma (Haim-Nachum et al., 2025), illustrating how treatment helped them cope. These interventions were compared to a same-length psycho-educational control video that presented generic information on stigma and mental health resources without the social contact elements. In a series of randomized controlled trials involving 3714 depressed adolescents and 1658 survivors of CT, we found significant reductions in stigma and increases in help seeking among those who viewed the intervention videos compared to controls (Haim-Nachum et al., 2025; Amsalem et al., 2024).

Recently, we sought to expand the outreach (i.e., increasing visibility) of these evidence-based brief video interventions by disseminating them on Instagram. We first tested the depression intervention video, evaluating youth engagement and help-seeking behaviors (Amsalem et al., 2024; Amsalem and Haim-Nachum, 2025). Specifically, we assessed the feasibility and acceptability of delivering this intervention via Instagram by comparing it to the psychoeducational control video across several key social media metrics (Lee et al., 2018): [1] Impressions, the number of times the ads were displayed; [2] Reach, the number of people who viewed the video; [3] Link clicks, the number of clicks to the ads' desired destination (i.e., mental health help); and [4] Recruitment cost-per-click (CPC), calculated by dividing ad costs by the total number of link clicks. Link clicks and CPC were primary outcomes, reflecting the audience's engagement and the campaign's cost-effectiveness. We found significant differences in link clicks to mental health resources and CPCs between those who viewed the depression intervention video and those who saw the psychoeducational control video, suggesting that such interventions have the potential to influence public attitudes toward stigma and mental health, and drive behavioral change among youth struggling with depression (Amsalem et al., 2024).

The current study aimed to expand the outreach (i.e., increasing visibility) and dissemination of evidence-based brief video interventions on Instagram to test youth engagement with the video and their help-seeking behaviors. Specifically, we sought to assess the feasibility and acceptability of delivering the CT-focused social contact-based intervention videos, both human-narrated and AI-generated videos, on Instagram, as compared to control videos. We hypothesized that individuals viewing the intervention videos (in both human-narrated and AI-generated formats) would exhibit higher link clicks to mental health resources and lower CPCs, indicating greater appeal and interest in the intervention videos compared to those viewing the control videos. This approach, which is the first to test such a delivery method for CT-focused content, allows us to assess help-seeking in a more ecologically valid manner with a large audience of youth.

## 2 Method

This Instagram study did not involve direct participant recruitment but instead disseminated a validated intervention via social media. No identifiable data were collected; only aggregated engagement metrics and demographic insights (e.g., age range) from Meta were analyzed. As no direct interaction or personal data collection occurred, individual consent and ethical approval were not required under standard research guidelines, a determination made following consultation with Yale University's Human Subjects Committee (see Declarations).

We launched two consecutive Instagram campaigns: an eight-day campaign in February and an eight-day campaign in March 2024. The first campaign featured a brief video intervention, proven effective in our previous crowdsourcing study, for reducing stigma and increasing help-seeking among survivors of CT (Haim-Nachum et al., 2025). As described above, this intervention video showcased an empowered young female describing her struggles with CT, including feelings of blame, shame, and guilt, misconceptions about treatment, and how social support from friends and professionals helped her overcome symptoms and recover. The control video was a same-length psycho-educational video that provided generic information and facts about signs, mental health resources, and approaches for seeking help, without personal narratives or social contact elements.

The second campaign included five videos: the same intervention and psychoeducational control videos mentioned above, along with two AI-generated videos featuring a young female avatar portraying the same content as the original human-based intervention. The AI-generated videos were produced using Colossyan Creator, an AI avatar video-generation platform (platform watermark visible in the video itself); the avatar was a young woman matching the demographic presentation of the human narrator. The script matched the human-narrated video verbatim, and on-screen text reading “AI generated video” was displayed continuously throughout each AI video, from the first frame to the last, disclosing the synthetic origin of the narrator to viewers. Additionally, there was an “About Me” control video featuring the same human actress describing her daily activities, with no mention of CT or mental health. This design choice aimed to test the hypothesis that personal narratives enhance viewer engagement by fostering identification and emotional connection—elements absent in the psychoeducational control video. The inclusion of AI videos allowed us to assess whether different presentation styles (AI vs. human narration) affect engagement and the effectiveness of conveying information about CT and reducing stigma. Video length ranged from 59 to 78 s across arms, per Meta Ads Manager records (Campaign 1: 78 s Intervention, 61 s Control; Campaign 2: 59 s AI-1, 78 s Control, 64 s Intervention, 61 s ‘About Me’ control, 64 s AI-2). The video links are available in Supplementary 1.

Meta was responsible for distributing the videos to youth in the target age range through its standard ad targeting mechanisms. Web-based advertisements in English were directed at youth Instagram users aged 18–24 currently living in the US. We utilized sponsored, geographically targeted ads on Instagram to encourage users to watch the brief videos. Instagram primarily operates on hashtag-based searches for users to explore content. It leverages algorithms to curate personal content feeds and provides a feed that prioritizes individual interests, the user's followers, the frequency of the user's posts, and the timing of content publication. Video ads contained the following hashtags: #childhoodtrauma, #fightingstigma, and virtual links that prompted youth to seek mental health help. Ads were displayed in a linear format, labeled as a sponsored ad within the user's personal Instagram feed. Captions were similar across all videos. Each ad included the option to “learn more” about available mental health resources by clicking on the link; this “Learn More” link was displayed continuously throughout each video alongside the campaign hashtags.

We conducted A/B testing to compare the performance of the above-described videos. A/B testing incorporates an embedded Meta Experiments tool allowing for the randomization of comparable data sets between the tested video ads while controlling for reach across different groups along with demographic elements (Kohavi et al., 2020). It helps ensure users will be evenly split and statistically comparable. Through A/B testing, it is possible to test how variable changes in social media algorithms perform to determine which one leads to better user engagement and satisfaction. Videos were randomly allocated to social media users. As with our previous depression study, outcome measures included Impressions, Reach, link clicks, and CPC rates (Amsalem and Haim-Nachum, 2025). Link clicks and CPCs are important social media metrics because they indicate the extent to which the audience finds the message appealing or interesting and act upon it (by clicking the links), determining the cost effectiveness (CPC) of the Instagram campaign.

## 3 Results

Regarding the feasibility and acceptability of brief social contact-based videos on social media, the two campaigns together yielded 996,924 impressions (i.e., the number of times the videos were displayed), reached 387,068 distinct viewers, and generated 6417 link clicks. The total cost of the two campaigns was $5343.75 ($3328.99 and $2014.76, for the first and second waves, respectively), which is similar to the budget range we typically allocate for crowdsourcing experiments.

The first campaign yielded 627,504 impressions, reached 210,756 Instagram users, and generated 4015 link clicks to mental health resources. A/B testing revealed that the intervention video garnered 2062 link clicks, while the psychoeducational control video received 1953 clicks. The cost-per-click (CPC) was lower for the intervention video at $0.81, compared to $0.85 for the control video. To account for unequal reach and impressions across arms, we calculated click-through rates (CTR) and compared them using two-proportion z-tests. On a per-reach basis, the intervention did not significantly outperform the control (1.89% vs. 1.92%; z = −0.56, p = .58); on a per-impression basis, the intervention significantly outperformed the control (0.68% vs. 0.60%; z = 3.72, p < .001). Refer to Table 1 for full A/B testing outcomes.

**Table 1:** A/B testing results for campaign 1.

Outcome	Intervention video	Psychoeducational control video
Impressions (n)	303,899	323,605
Reach (n)	109,156	101,600
Link Clicks (n)	2062	1953
Cost Per Click (CPC, $)	0.81	0.85

The second campaign generated 369,420 impressions, reached 176,312 users, and resulted in 2402 link clicks, reinforcing the feasibility and acceptability of brief social contact-based videos on Instagram (Table 2). The CPC for the intervention video was $0.79, compared to $0.89 for the psychoeducational control video and $0.92 for the ‘About Me’ control video. Click-through rates (CTR), compared via two-proportion z-tests, showed that the intervention significantly outperformed the psychoeducational control on a per-impression basis (0.67% vs. 0.57%; p = .010) but not significantly on a per-reach basis (1.35% vs. 1.24%; p = .17), and significantly outperformed the ‘About Me’ control on both bases (per-reach: 1.35% vs. 1.16%, p = .014; per-impression: 0.67% vs. 0.57%, p = .010). The AI-generated videos (CPC: $0.77 for AI-1, $0.84 for AI-2) did not merely perform similarly to the human-narrated intervention: per-reach CTR was significantly higher for both AI arms than for the intervention (AI-1: 1.56% vs. 1.35%, p = .020; AI-2: 1.55% vs. 1.35%, p = .031). As a complementary acceptability indicator, Meta Ads Manager also reports hook rate (percentage of viewers retained past the first three seconds) and hold rate (percentage retained near the end of the video) for each ad; across arms these ranged from 7 to 18% and 0.3–13.4%, respectively, with no consistent pattern distinguishing human-narrated from AI-narrated videos. See Table 2 for full A/B testing outcomes.

**Table 2:** A/B testing results for campaign 2.

Outcome	Intervention	AI 1	AI 2	Psychoeducational control	‘About Me’ control
Impressions (n)	76,035	69,614	66,733	79,675	77,363
Reach (n)	37,575	33,436	30,924	36,473	37,904
Link clicks (n)	509	523	480	452	438
Cost per click (CPC, $)	0.79	0.77	0.84	0.89	0.92

## 4 Discussion

This study aimed to assess the feasibility and acceptability of delivering evidence-based brief video interventions related to childhood trauma through Instagram, focusing on engagement and help-seeking behaviors among youth. We measured key social media engagement outcomes across two consecutive Instagram campaigns, including impressions, reach, link clicks to mental health resources, and cost-per-click (CPC), to determine the effectiveness and cost-efficiency of the intervention videos compared to control videos. Additionally, we sought to replicate these findings and explore the feasibility of additional videos, including those generated by artificial intelligence (AI).

Our findings indicate high engagement rates, with a combined total of 996,924 impressions, 387,068 unique viewers, and 6417 link clicks to mental health resources. Intervention videos – whether human-narrated or AI-generated – generally outperformed the control videos on click-through rate, though this held more consistently on a per-impression than a per-reach basis (significant in Campaign 2 against the ‘About Me’ control but not against the psychoeducational control, and not significant in Campaign 1 per-reach), and AI-generated videos showed significantly higher per-reach CTR than the human-narrated intervention. These findings underscore Instagram's potential as a cost-effective platform for disseminating childhood trauma-related brief stigma-reducing interventions to large youth audiences, aligning with public health goals to extend evidence-based interventions beyond laboratory settings. To our knowledge, this is the first study to assess the feasibility and acceptability of CT-related brief online video interventions among youth on Instagram.

The total cost of the campaigns ($5343.75), which is comparable to expenses incurred in crowdsourcing platform experiments (Amsalem and Martin, 2022; Amsalem et al., 2024), yielded a much higher outreach of 387,068 viewers. This underscores the extensive dissemination and potential impact that social media can achieve. Implementing an intervention initially tested in a controlled laboratory environment (i.e., crowdsourcing platforms) aligns with the Surgeon General's Advisory (Office of the Surgeon General, 2023), which recommends using safe content in real-world social media settings, as reflected in the high outreach numbers. Testing content in virtual laboratories beforehand minimizes the risks associated with launching untested interventions on social media, thereby safeguarding the well-being of young audiences. While further research is necessary to assess the long-term impact, our cost-effective Instagram campaign demonstrates the practicality and effectiveness of utilizing social media platforms for implementing safe, evidence-based mental health interventions.

Although our study did not explicitly target or screen for survivors of CT, the high prevalence of CT reported in the literature (Brown et al., 2023; Merrick et al., 2018), combined with our algorithmic targeting using CT-related hashtags and content preferences, likely increased our reach to youth who express interest in CT-related content, know someone dealing with CT, or experience it firsthand. The substantial engagement metrics –approximately 997,000 impressions and a reach of 387,000–support the likelihood that our campaign connected with young CT survivors or those whose peers have experienced CT-related events. Furthermore, our sampling method has the potential to influence a wider audience utilizing the platform for educational and public health purposes.

The mechanism of action in our interventions is rooted in the use of social contact-based elements, which foster identification and emotional engagement (Shahwan et al., 2022). Previous studies conducted in controlled environments have demonstrated that social contact-based approaches lead to a significant reduction in stigma and an increase in self-reported help-seeking intentions (Haim-Nachum et al., 2025; Amsalem et al., 2024). Building on our recent work with depressed youth (Amsalem et al., 2024), our current findings extend the understanding of CT-related stigma and treatment-seeking behavior to real-world settings by investigating the engagement and behavioral impact of these interventions when disseminated through social media. High engagement metrics (i.e., impressions and reach) of videos incorporating social contact elements (Intervention, AI-generated videos, and Control 2) support this theory. The intervention and AI-generated videos yielded higher link clicks for treatment, highlighting the effectiveness of social contact-based intervention videos. While the “About Me” control video was expected to be engaging due to its personal content, it was not anticipated to promote help-seeking behavior, as it was unrelated to mental health, CT, or stigma, underscoring our findings. Notably, viewers of the AI-generated videos were explicitly told the narrator was synthetic – on-screen text disclosing this was visible throughout each AI video – yet engagement with these videos matched or exceeded engagement with the human-narrated video. Because the synthetic origin of the narrator was disclosed rather than concealed, this is not a case of a fabricated, undisclosed testimony outperforming a genuine one; rather, it suggests either that disclosed AI narration did not measurably undermine credibility or identification for this audience, or that engagement at the ad level (a click) reflects content and framing more than perceived authenticity, a distinction that may not hold at deeper levels of engagement (e.g., actual help-seeking behavior after the click), which our data cannot address. We frame this as a question for future research rather than a resolved finding. We also note that these campaigns were run in 2024, and that AI avatar and voice-generation quality has advanced considerably since; findings regarding disclosed AI narration may not generalize to more recent tools. Leveraging evidence-based interventions through social media channels can provide a significant public health opportunity to reduce stigma and promote mental health care for those in need and their peers (Mheidly and Fares, 2020).

Our findings contribute to the growing body of evidence indicating that social media interventions can facilitate access to mental health care and drive positive changes among youth (Baskerville et al., 2015), particularly by generating link clicks to mental health resources (Plackett et al., 2023). These findings build upon our previous evidence regarding the interventions' effectiveness in increasing individuals' self-reported openness to treatment seeking (Haim-Nachum et al., 2025; Amsalem et al., 2024), suggesting potential efficacy in enhancing the outreach and impact of existing interventions and promoting mental health behaviors. Given that our results represent an initial level of treatment-seeking behavior, future studies should monitor and follow up on such behaviors. For example, implementing landing pages or web portals could help track individuals' treatment receipt, continuity, and effectiveness, thus assessing the long-term effects of this intervention.

### 4.1 Limitations

Further research is needed to monitor and evaluate actual long-term help-seeking behaviors following individuals' engagement with this intervention. Reach reflects distinct viewers within each campaign, guaranteed by Meta's split-test assignment; we cannot rule out overlap in viewers between the two campaigns, which were run separately in February and March 2024. Moreover, we cannot characterize who was reached, since no audience-level demographic or clinical data were collected; recruitment source is known to shape who arrives in an internet intervention, both in demographics and clinical severity (Lindner et al., 2015). Additionally, due to the nature of this real-world campaign, we were unable to gather comprehensive demographic data, nor did we collect relevant information such as prior or current treatment details, stigma scores, or “likes,” which could offer a further indicator of engagement; we will ensure such metrics are systematically collected in future campaigns. Given previous findings that highlight the importance of tailoring brief video interventions to specific audience demographics (e.g., gender, race, ethnicity), future social media videos should be designed to cater to a more diverse audience (Amsalem et al., 2022). Finally, we focused exclusively on Instagram; future studies should explore other social media platforms, such as TikTok.

### 4.2 Conclusions

In summary, our research contributes to the growing understanding of how social media can be leveraged for youth mental health, specifically concerning CT. The application of our social contact-based brief video interventions in a controlled environment before disseminating them on Instagram has shown promising results in stigma reduction and mental health outreach, extending our prior studies on depression to another common phenomenon of CT. This endeavor demonstrates the practical potential efficacy of such interventions in engaging youth struggling with CT and encouraging behavioral change. While there remains much to explore, our findings offer valuable insights into future research and interventions aimed at promoting the safety and well-being of youth in the digital realm, particularly those grappling with the consequences of CT and their peers. Our evolving approach represents a significant step toward understanding and balancing the potential benefits and risks of social media for young users, providing more support for its impact.

## Consent for publication

Not applicable.

## Ethics approval and consent to participate

This study did not involve human participants, identifiable data, or biological material. Instead, it relied exclusively on aggregated engagement metrics collected through Meta's Ads Manager for Instagram, such as impressions, reach, and link clicks. No direct contact with users occurred, and no individual-level data were accessed or stored. In line with institutional and international ethical standards for public data analysis, ethical approval was not required. Prior to conducting the study, we consulted with Yale University's Human Subjects Committee regarding the nature of the project; based on that consultation, the project was determined not to require IRB review because it involved only aggregated, de-identified campaign metrics and did not involve interaction with human participants or access to identifiable individual-level data. The “Learn More” link on each ad directed users to information about available mental health resources. The brief video intervention disseminated in this study had itself previously been shown effective in reducing stigma and increasing help-seeking among survivors of childhood trauma in a controlled crowdsourcing study, approved by the New York State Psychiatric Institute Institutional Review Board [No. 8453; (Haim-Nachum et al., 2025)], which provided additional assurance regarding participant safety prior to this dissemination study.

## Funding

This project was partially supported by an unrestricted educational grant from Meta, which provided the ad credits that enabled the campaign. However, Meta had no involvement in the design, execution, analysis, or reporting of the study.

## Declaration of competing interest

The authors declare that they have no known competing financial interests or personal relationships that could have appeared to influence the work reported in this paper.

## Data Availability

All aggregated engagement data analyzed in this study are included in the article and its supplementary material. Video content used in the campaigns is publicly available and linked in the Supplementary Material 1 document.

## Appendix A Supplementary data

Supplementary 1 Videos included in the Instagram campaigns.
