# Supplementary material for: Disseminating brief video-based mental health interventions on Instagram: Reach, engagement, and real-world implementation
Source: Internet Interv. 2026 Aug 12;45:100990. doi: 10.1016/j.invent.2026.100990 (PMC13488557; doi:10.1016/j.invent.2026.100990)
Supplement: Supplementary file 1 [file mmc1.docx]

***Supplementary 1: Videos included in the Instagram campaigns***

**Intervention video** <https://youtu.be/n2KQgSciHds>

**AI video 1:** <https://youtu.be/S-wa-F2b3RA>

**AI video 2:** <https://youtu.be/7ATqcY4uovw>

**Psychoeducational control video** <https://youtu.be/vkqTGm_M4R4>

**‘About Me’ control video:** <https://youtu.be/58QPto-KQls>
